# From Throat to Limb: A Novel Case of Compartment Syndrome Following Group A Streptococcal Pharyngitis

**DOI:** 10.1155/crdi/5016797

**Published:** 2025-05-06

**Authors:** Simar Goyal, Justin Choy, Kendall Wermine, Sidart Pradeep, Khazeema Hafeez, Rusty Milhoan, Lavanya Srinivasan

**Affiliations:** ^1^Anne Burnett Marion School of Medicine at Texas Christian University, 1100 W. Rosedale St., Fort Worth, Texas, USA; ^2^Baylor Scott & White All Saints Medical Center, 1400 8th Ave, Fort Worth, Texas, USA

**Keywords:** case report, compartment syndrome, Group A Streptococcus, myositis, pharyngitis

## Abstract

Acute compartment syndrome is a medical emergency caused by increased pressure within a closed fascial space, leading to tissue ischemia and potential limb loss or death if untreated. While typically secondary to trauma, rare cases have been associated with streptococcal infections. However, no documented case exists of compartment syndrome originating from Group A Streptococcus (GAS) pharyngitis. We present a 35-year-old female, with prediabetes, who presented to the emergency department with worsening right lower extremity (RLE) pain and edema following a febrile illness with pharyngitis. Throat swab on admission and subsequent blood cultures tested positive for beta-hemolytic GAS. Despite initial management for sepsis and cellulitis with myositis, she developed compartment syndrome requiring urgent fasciotomy. Her postoperative course was complicated by hypotension, toxic shock-like syndrome, menorrhagia, and transaminitis, but she ultimately recovered with IV antibiotics and stepwise Jacob's Ladder surgical wound closure. This report serves as the first known case of nontraumatic acute lower extremity compartment syndrome derived from disseminated GAS pharyngitis and may point to the development of novel virulence factor(s) for emerging strains of GAS in the United States. The case underscores the importance of recognizing GAS pharyngitis as a potential source of severe systemic infections, as early identification and aggressive management of invasive GAS infections may help prevent life-threatening complications.

## 1. Introduction

Acute compartment syndrome is a surgical emergency, resulting from increased pressure within a closed fascial space leading to tissue ischemia and potential tissue necrosis if not promptly treated [1, 2]. While this diagnosis is commonly associated with trauma, rare cases have been linked to Group A Streptococcus (GAS) infections, particularly following cellulitis or invasive soft tissue infections [3–8]. GAS is a Gram-positive bacterium responsible for a spectrum of infections, ranging from mild pharyngitis to severe invasive diseases such as necrotizing fasciitis, streptococcal toxic shock syndrome, and bacteremia [9]. Although GAS is a well-known cause of deep tissue infections, there have been no reported cases of GAS pharyngitis progressing to acute compartment syndrome. We present the first known case of nontraumatic acute lower extremity compartment syndrome derived from disseminated GAS pharyngitis.

## 2. Case

Our patient is a 35-year-old prediabetic female, with Hemoglobin A1c of 5.8 and no other pertinent medical, family, or social history, who presented to the emergency department with worsening right lower extremity (RLE) pain and edema with onset one day prior to presentation. She initially presented to her primary care provider earlier that day and was recommended to present to the emergency department for treatment. She denied trauma, scratches, falls, or open wounds to the RLE. Prior to the presentation, the patient endorsed a maximum temperature of 103F, chills, sore throat, body aches, and night sweats for 2–3 days. Rapid GAS throat swab taken at presentation to the emergency department tested positive for the presence of GAS. Moreover, two sets of blood cultures were drawn on the presentation 3 h apart. Both sets grew penicillin susceptible beta-hemolytic GAS detected by Verigene molecular assay and were isolated by culture.

**Figure 1:**
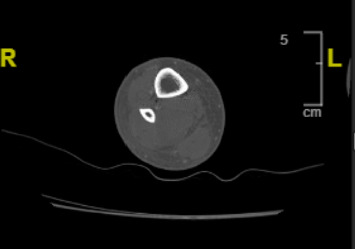
Subtle edema in the muscles in the mid-distal posterior calf reflecting possible myositis.

The patient's vitals on admission were significant for normothermia, tachycardia ranging from 112 to 131 beats per minute, with a systolic blood pressure ranging from 113 to 134 mmHg, and a diastolic blood pressure ranging from 84 to 92 mmHg. Her physical exam showed 1-2+ pitting edema of the RLE below the patella, and she reported pain upon palpation of the right calf. There was no overlying erythema or crepitus. Complete metabolic panel was remarkable for elevated AST of 74 U/L, elevated ALT of 104 U/L, elevated creatinine to 1.4 mg/dL from baseline 0.58 mg/dL, and mild hypokalemia of 3.5 mEq/L from a baseline of 3.9 mEq/L. WBC were elevated to 23,000/L and Hgb was 10.0 g/dL from unknown baseline. Creatine kinase (CK) was 463 U/L on admission. Notably, RLE ultrasound performed in the emergency department was negative for deep vein thrombosis. Computed tomography (CT) scan of the RLE with contrast was notable for subcutaneous edema, and the radiologist noted concern for cellulitis and myositis without abscess formation (Figure 1). The patient was admitted for sepsis secondary to cellulitis with associated myositis and was started on broad spectrum antibiotics with vancomycin, cefepime, and metronidazole with IV normal saline at 150 mL/hr.

On hospital Day #2, the patient reported worsening RLE pain with physical exam notable for a tense, swollen anterior compartment of the leg. Surgery was consulted, and the patient was urgently brought to the operating room due to acute compartment syndrome with myositis and rhabdomyolysis, evidenced by repeat labs showed CK 2,076 U/L and +3 blood on urinalysis. A two-incision fasciotomy was performed on the RLE below the knee, and a small fasciotomy was made to the right thigh to assess for compartment syndrome. First, a longitudinal incision above the lateral malleolus was made using a 15-mm blade. Then, a 10-cm longitudinal incision was made over the anterior muscle compartment midway between the tibia and fibula. The anterior compartment fascia was opened with blunt dissection, resulting in significant pressure release. The lateral fasciotomy incision was extended down to the ankle, and the posterior compartment fascia was opened. Then, an 8-cm longitudinal incision was made on the medial surface of the lower leg 2 cm medial to the tibia. Once the leg fasciotomies were completed, a 10-cm incision was made on the lateral thigh. The muscle was deemed edematous but viable with no evidence of compartment syndrome. The area was irrigated and packed with 4 × 4 gauze soaked in Vashe.

Following the procedure, the patient was admitted to the wards for monitoring. On hospital Day #3, she became hypotensive with a blood pressure of 70/40 mmHg that was nonresponsive to a bolus of 1 L of normal saline. She was diagnosed with Toxic shock-like syndrome secondary to GAS and was transferred to the ICU for closer monitoring, given her hemodynamic instability. Despite her hypotension, the patient maintained a normal heart rate and normal respiration rate. She received 3 L of lactated ringers and was started on a 14-day course of IV cefazolin 2 g in sodium chloride 0.9% 50 mL and six doses of clindamycin 900 mg/50 mL per infectious disease consult. A transthoracic echocardiogram showed normal biventricular functions without valvular vegetations. Upon regaining hemodynamic stability, the patient was transferred from the ICU to the wards on the same day. Pressors were not required to maintain normotension.

**Figure 2:**
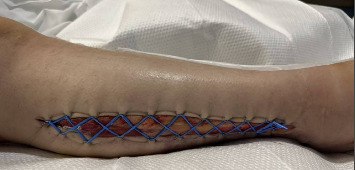
Jacob's Ladder closure for lower leg fasciotomy.

On hospital Day #4, the patient underwent RLE wound exploration and partial closure of wounds. No signs of necrosis were noted in the right leg or calf wounds. Right thigh wound was closed using 3-0 nylon suture in simple interrupted fashion, and the medial right calf was closed with 3-0 nylon suture in vertical mattress suture. Then, the lateral right calf fasciotomy incision was partially closed by placing a mini blue vessel loop in Jacob's Ladder formation using 11 staples per side (Figure 2). Daily cinching of the Jacob's Ladder was performed at bedside for 8 days. On hospital Day 12, the patient underwent wound exploration and partial complex closure of the right lateral wound. Significant edema in the RLE made reapproximation challenging. The wound and skin were closed in three layers, with the lower portion near the ankle left open due to persistent edema. A 10-French Jackson–Pratt drain was placed posteriorly.

Following subsequent closure of the fasciotomy, the patient completed her initial 14-day IV cefazolin course. Per recommendations from the infectious disease service, the patient was initiated on a 7-day course of oral cephalexin. The patient remained stable and afebrile for the remainder of her hospitalization. Repeat blood cultures obtained postoperatively on hospital Day 3 showed no growth.

On hospital Day #17, the patient had an elevated AST of 173 U/L and an elevated ALT of 278 U/L, prompting a hepatology consultation. Abdominal ultrasound showed hepatic steatosis. The patient's viral panel was negative to hepatitis A, hepatitis B, hepatitis C, and hepatitis C RNA. She tested positive for smooth-muscle antibody. Per hepatology recommendations, hepatotoxic medications were discontinued.

The remainder of the patient's hospital course was uneventful aside from the occurrence of heavy menstrual bleeding and associated iron deficiency anemia (Hgb 8.5) on hospital Day #10, which resolved with norethisterone 5 g BID. Pelvic ultrasound revealed an endometrial stripe measuring 16 mm with slightly heterogeneous echogenicity/echotexture, findings suggestive of a blood clot.

The patient was deemed stable and discharged from the hospital despite persistent elevated transaminases (AST 169 and ALT 304) on hospital Day #18. At discharge, the patient endorsed intact sensation to the lower extremity and was able to ambulate with minimal assistance. The patient had two outpatient follow-ups with hepatology after discharge and now follows as needed.

**Figure 3:**
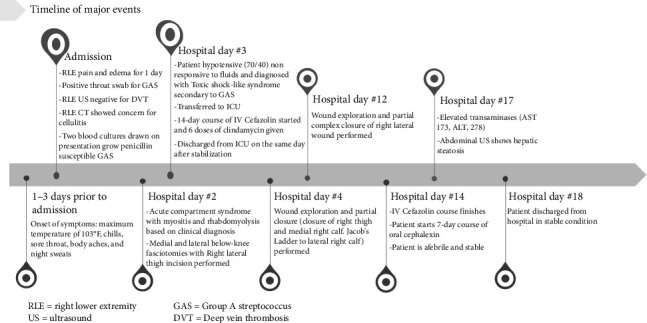
Timeline of major events.

A synopsis of this patient's treatment course can be found below (Figure 3).

## 3. Discussion

GAS is a Gram-positive bacterium responsible for a range of human infections, including pharyngitis, impetigo, streptococcal toxic shock-like syndrome, bacteremia, and necrotizing fasciitis [9]. GAS pharyngitis is particularly common, with an estimated 288.6 million cases annually in children aged 4–15 years. In contrast, GAS bacteremia is far less frequent, with approximately 600,000 cases reported each year [10]. Previous reports have documented cases of compartment syndrome associated with streptococcal infections, typically following trauma or cellulitis [3–8]. However, no prior cases have identified GAS pharyngitis as a potential source of the subsequent compartment syndrome. This case is unique in that it describes a nontraumatic acute lower extremity compartment syndrome resulting from disseminated GAS pharyngitis, confirmed by a positive throat swab and two sets of blood cultures.

Compartment syndrome usually manifests after a trauma especially crush injuries. It was highly unusual for the patient to have had no previous open wounds or injuries to the site, as nontraumatic compartment syndrome due to GAS is an uncommon medical pathology. The combination of her unusual etiology for compartment syndrome in combination with the overlap between her symptoms and the classic presentation of deep vein thrombosis resulted in delays to both presentation and definitive surgical treatment in this case.

There is substantial diagnostic evidence supporting GAS pharyngitis as the primary source of infection leading to bacteremia, myositis, and subsequent compartment syndrome. The initial rapid antigen detection test (RADT) for GAS pharyngitis, which was used as a screening tool in this patient, is highly specific (∼95%) but has variable sensitivity ranging from 70% to 99% [10]. Bacteremia was confirmed using the Verigene molecular assay and traditional culture methods, both of which identified penicillin-susceptible beta-hemolytic GAS. The Verigene molecular assay, which detects GAS in blood cultures, has a reported sensitivity of 98.6% and specificity of 99.5% [11], further reinforcing the diagnosis.

Given the strong diagnostic evidence linking GAS pharyngitis to the subsequent bacteremia and compartment syndrome in our patient, it is important to contextualize this case within the existing literature. While GAS is a well-known pathogen capable of causing invasive infections, reports of nontraumatic compartment syndrome secondary to GAS bacteremia remain rare. Understanding the clinical patterns and outcomes of such cases can provide valuable insight into the severity of this condition and the critical need for early recognition and intervention.

A study by Kleshinski et al. found 13 cases of nontraumatic compartment syndrome secondary due to GAS from the period of 1950–2007 [4]. The average age of the cohort was 34 years, and 77% of the patients had no significant medical history or evidence of immunosuppression, similar to our patient. All 13 reported patients required surgical intervention with debridement and/or fasciotomy, with an overall mortality rate of 15%. This is not an uncommon result for compartment syndrome. A larger multicenter study that analyzed nontraumatic extremity compartment syndrome of various etiologies found an in-hospital mortality rate of 20% [12]. Taken together, it can be reasonably believed that our patient would have suffered limb or life-threatening complications without intervention. These findings demonstrate the importance of prompt medical and surgical interventions upon recognition of the disease process to prevent complications and death.

The growing recognition of GAS as a cause of severe, invasive infections, including compartment syndrome, raises important concerns about its evolving epidemiology and pathogenicity. While historical data provide insight into the severity and outcomes of GAS-associated compartment syndrome, recent trends suggest an increased incidence of disseminated GAS in the United States, Canada, and Europe in recent years [13]. In the absence of a vaccine, treatment for GAS has been centered around antibiotic usage, with emphasis on beta-lactam antibiotics. In turn, this has propelled mutation and the emergence of novel virulence factors and disease processes for the bacterium [11]. Given these recent increases in GAS bacteremia, our case may reflect some early signs of a changing virulence pattern of GAS. However, a notable limitation of our study is the lack of microbiological analysis of the GAS strain that infected our patient. This analysis could have potentially provided insight of particular virulence factors that lead to the development of the compartment syndrome. As a result, we are unable to determine whether specific strain-related or genetic features may have played a role in this aggressive clinical presentation.

While the focus of our case has been on the pathogenesis and clinical course of GAS-associated compartment syndrome, the management of such cases extends beyond infection control to include surgical considerations. One critical aspect of postoperative care is wound closure following fasciotomy, a procedure that presents unique challenges due to tissue edema and skin contracture.

Wound closure after fasciotomies is a controversial topic with variable factors such as availability of resources and experience swaying providers toward one technique over another. Immediate primary closure is not usually possible due to the tissue edema and skin contracture present [14, 15]. Therefore, split-thickness skin grafts (STSG) are often employed as an alternative. However, STSG has been associated with increased rates of morbidity, scarring, infection, pain at graft site, and poor cosmetic outcomes [16, 17]. In the case of our patient, a vessel loop in a Jacob's Ladder formation was used to close the lateral leg wound. Cohn et al. initially proposed the use of vessel loops and skin staples for delayed primary closure of fasciotomy wounds, which decreased the risks associated with STSG while allowing for a more cosmetically appealing closure without a lengthened hospital stay [18]. Notably, this technique is typically inexpensive due to the availability of the materials used [19].

It is necessary to have early surgical and medical management with prompt Gram-positive antibiotic treatment in cases of nontraumatic compartment syndrome secondary to disseminated GAS infection. We recommend careful physical examination and urgent fasciotomies when clinical indications exist.

## 4. Conclusions

This case highlights a previously unreported progression of GAS pharyngitis leading to acute compartment syndrome. While GAS is a well-documented cause of invasive infections, its role in nontraumatic compartment syndrome remains rare. This case underscores the potential for emerging GAS virulence patterns and the need for heightened clinical awareness. Early recognition and prompt treatment of GAS infections are crucial to preventing severe complications, including life-threatening soft tissue and systemic involvement.

## Data Availability

Data sharing is not applicable to this article as no new data were created or analyzed in this study.
